# Impact of the Physical Activity Environment on Change in Body Mass Index Percentile in Child Care Centers Serving Children with Disabilities

**DOI:** 10.3390/nu16152457

**Published:** 2024-07-29

**Authors:** Martha H. Bloyer, Ruixuan Ma, Yaray Agosto, Carolina Velasquez, Katheryn Espina, Joanne Palenzuela, Michelle Schladant, Julieta Hernandez, Sarah E. Messiah, Ruby Natale

**Affiliations:** 1 Department of Physical Therapy, University of Miami School of Medicine, Coral Gables, FL 33146, USA; mhb79@med.miami.edu; 2 Department of Biostatistics, University of Miami School of Medicine, Miami, FL 33136, USA; rxm1424@med.miami.edu; 3 Department of Pediatrics, Mailman Center for Child Development, University of Miami School of Medicine, Miami, FL 33136, USA; yagosto@med.miami.edu (Y.A.); kxe258@med.miami.edu (K.E.); jpalenzuela@miami.edu (J.P.); mschladant@med.miami.edu (M.S.); jhernand@med.miami.edu (J.H.); 4 University of Texas Health Science Center at Houston School of Public Health, Center for Pediatric Population Health, Houston, TX 77030, USA; sarah.e.messiah@uth.tmc.edu; 5 Department of Pediatrics, McGovern Medical School, University of Texas Health Science Center at Houston, Houston, TX 77030, USA

**Keywords:** children, children with disabilities, obesity, physical activity, percentile body mass index, early childhood

## Abstract

Childcare centers (CCCs) can provide opportunities to implement physical activity (PA) via health promotion interventions to prevent obesity and associated chronic disease risk factors in young children. This study evaluated the impact of the Healthy Caregivers-Healthy Children (HC2) intervention on body mass index percentile (PBMI) and the PA environment in CCCs serving children with disabilities (CWD) over one school year. Ten CCCs were cluster-randomized to either (1) an intervention arm that received the HC2 curriculum adapted for CWD or (2) an attention control arm. Mixed-effect linear regression models analyzed the relationship between change in child PBMI and CCC childcare center PA environment by experimental condition and child disability status over one school year. Findings showed a significant decrease in PBMI among children in the HC2 centers (−6.74, *p* = 0.007) versus those in control centers (−1.35, *p* = 0.74) over one school year. Increased PA staff behaviors (mean change 3.66, *p* < 0.001) and PA policies (mean change 6.12, *p* < 0.001) were shown in intervention centers during the same period. Conversely, there was a significant increase in sedentary opportunities (mean change 4.45, *p* < 0.001) and a decrease in the portable play environment (mean change −3.16, *p* = 0.03) and fixed play environment (mean change −2.59, *p* = 0.04) in control centers. No significant differences were found in PBMI changes between CWD and children without disabilities (beta = 1.62, 95% CI [−7.52, 10.76], *p* = 0.73), suggesting the intervention’s efficacy does not differ by disability status. These results underscore the importance of (1) including young CWD and (2) PA and the supporting environment in CCC health promotion and obesity prevention interventions.

## 1. Introduction

Reducing childhood obesity is a health priority, given globally an estimated thirty-nine million children under the age of five years are overweight or obese [1]. In the United States (US) one in five children and adolescents have obesity [2]. Pediatric obesity is associated with numerous cardiometabolic diseases [3] and musculoskeletal risk factors that track strongly into adulthood. Specifically, obesity in childhood is a precursor to type 2 diabetes, cardiovascular disease, stroke, osteoarthritis, certain types of cancer, COVID-19 illness, depression, and anxiety making early childhood a crucial window for healthy weight development [4,5,6].

Children with disabilities (CWD), racial/ethnic minority groups, and those from low socioeconomic status (SES) backgrounds are disproportionately impacted by obesity, yet this intersection (disability/race/ethnicity/low SES) has rarely been studied, especially in early childhood [2,7,8]. Specifically (1) CWD (those with developmental, physical, or intellectual disabilities with a particular emphasis on those with developmental disabilities as defined by McPherson et al. [8,9]) have a 38% higher prevalence of obesity compared to their peers without disabilities [10]; (2) non-Hispanic Black and Hispanic preschool-aged children have a higher prevalence of obesity compared to their non-Hispanic White counterparts [1,2]; and (3) low SES background is a significant social determinant of health (SDOH) that drives obesity disparities in young children [1,2,11]. Despite these disparities, CWD are typically not included in behavioral and medical interventions promoting a healthy lifestyle during the early childhood years [7].

Almost two-thirds of children under 5 years spend time in early care and education [12]. As such, the childcare center (CCC) environment can provide opportunities to implement physical activity (PA) and health promotion interventions to reduce chronic disease risk factors associated with obesity at a young age [13,14,15,16,17,18]. Others have concluded there is a need to encourage/support active behaviors in early-learning settings, particularly for children in center- and home-based childcare [19]. Through multiple randomized control trials over the past 15 years, our team has developed Healthy Caregivers, Healthy Children (HC2), an obesity prevention intervention developed specifically for the CCC environment. HC2 has shown positive health and wellness outcomes, including the maintenance of healthy weight trajectories among racially and ethnically diverse children from low-SES backgrounds [20,21]. The aims of this study were to evaluate changes in (1) the CCC physical activity PA environment and (2) child body mass index percentile (PBMI) by experimental condition and child disability status over one school year.

## 2. Materials and Methods

### 2.1. Study Design Overview

This study included analysis of data across one school year and two time points; October–December 2021 (T1, beginning of school year) and May–June 2022 (T2, end of school year). These analyses are from a cluster randomized controlled trial (ClinicalTrials.gov ID: NCT05106426) of HC2, adapted for CWD. Team members for this study consisted of an education specialist, a nutritionist, a pediatrician, a physical therapist, a psychologist, a pediatric epidemiologist, statisticians, and research assistants. The University of Miami Institutional Review Board approved the study protocol.

A total of ten (10) inclusion-based CCCs serving CWD were randomized to one of two arms: (1) an *Intervention Arm* that received the HC2 curriculum adapted for CWD, which consisted of environmental/center modifications (i.e., menu changes) and the evidence-based HC2 role modeling/gatekeeper curriculum for parents and teachers; or (2) a *Control Arm* that received the attention control ‘Jump Start’, a behavioral support consultation program. All pre–post measures and incentives were the same across both study arms.

### 2.2. Inclusion and Exclusion Criteria

CCC inclusion criteria were as follows: (1) Enrollment of ≥30 children ages 2-to-5 with a diagnosis or risk for disability as measured by appropriate diagnostic instruments and procedures and informed clinical opinion; (2) Serve low-income families; and (3) Reflect the racial–ethnic diversity of Miami Dade County, Florida (69.1% Hispanic or Latino, 17.1% Black or African American, and 13.8% non-Hispanic White) [22,23]. Children with disabilities were identified if they had an Individualized Education Plan (IEP) or an Individualized Family Service Plan (IFSP) or a failed screening on the Ages and Stages Questionnaire indicating risk for disability. Child exclusion criteria consisted of (1) non-consent by a parent to participate; and (2) children with feeding tubes or those that brought their own meals due to dietary restrictions or classified as ‘failure to thrive’ and, thus, could have difficulty participating in the physical activity programming.

### 2.3. Study Procedures

CCCs that met inclusion criteria were contacted via a telephone call. After explanation of the HC2 randomized cluster trial, participating CCC directors and teachers provided written signed consent. Parents/guardians were recruited via flyers and announcements. A total of 274 caregivers/parents provided written consent to participate in the study and completed baseline survey packets.

To ensure consistent study execution and adherence, research assistants completed mandatory training focusing on previous HC2 research protocols and methodologies. Training included on-site activities and role-playing scenarios addressing potential biases related to obesity prevention among children with disabilities. Research assistants followed an implementation protocol within the CCC, monitored by program managers, through evaluations during the start of the study and one month later. Research assistants facilitated communication with caregivers during drop-off and pick-up times at the CCCs to establish contact. In-person contact procedures adhered to the COVID-19 distancing guidelines and procedures set at each CCC. During these interactions, parents/caregivers were provided consent forms, completed the surveys, or were provided the packets to be completed at home. Survey packets were available in English and Spanish.

For the intervention arm, the HC2 toolkit was implemented on a weekly basis for 1 h in each center throughout the school year. The HC2 toolkit consists of a bilingual obesity prevention curriculum that includes 42 lesson plans and materials designed to incorporate current nutrition and physical activity policy requirements for preschool children in Florida. The four foundational policies of the HC2 toolkit include (1) snack policy, (2) beverage policy, (3) physical activity policy, and (4) screen time policy [13]. The HC2 toolkit has been shown to be effective in maintaining a healthy body mass index, improving the consumption of healthy foods (fruits and vegetables), decreasing the consumption of unhealthy foods, and increasing physical activity among racially and ethnically diverse preschool age children from low socioeconomic backgrounds [13].

HC2 modifications were made to accommodate CWD based on disability type. For example, children with orthopedic impairments were provided with adaptive equipment or adaptive switches to access the environment that supported their gross or fine motor delays. Children with intellectual disabilities were provided with picture-supported guides for learning or provided with repetitive routines to allow mastery of a skill. Children with autism spectrum disorder (ASD) were provided with sensory activities (i.e., they could touch and smell food as a first step in trying new foods), and children with sensitivities were given opportunities to participate in activities that assisted in the prevention of isolation on the playground.

The control arm received an attention control consisting of Jump Start, a behavioral support program that has been in existence for 4 years and has targeted CWD; therefore, adaptations were not needed [24].

### 2.4. Outcome Measures

#### 2.4.1. Child Body Mass Index Percentile (PBMI) (Primary Outcome)

Once consent and caregiver demographic forms were received, collection of child’s height and weight were obtained. Research assistants collected these data after receiving training and observation of correct measurements that adhered to the guidelines set by the Centers for Disease Control and Prevention (CDC) [25]. Height (cm) was measured using a Health o Meter portable stadiometer model 221HR and weight (kg) was measured using a Seca Clara 803 digital scale. All height and weight data were converted to age- and sex-adjusted percentiles (PBMIs). Specifically, PBMI is used to measure a child’s weight in relation to their height adjusted for age and sex [5,26]. PBMI weight status categories for children are as follows; less than the 5th percentile (underweight), 5th to 84th percentile (healthy weight), 85th to <95th percentile (overweight), and ≥95th percentile (obesity) [5,26].

#### 2.4.2. Environmental and Policy Outcomes (EPAOs) (Primary Exposure)

The Environmental and Policy Outcomes (EPAOs) is a tool designed to evaluate practices, environmental attributes, and policies of early care and education settings that influence children’s nutrition, physical activity, and sedentary environments [27,28]. EPAO categories include (1) Eating Occasions—Foods, (2) Eating Occasions—Beverages, (3) Eating Occasions—Staff Behaviors, (4) Physical Activity—Child Behaviors, (5) Sedentary Activity—Child, (6) Physical Activity—Staff Behavior, and (7) Center Environment. This study focused on physical and sedentary activity—child and staff behaviors, and center environment and policies related to PA. Through full one-day observations at each CCC location, the EPAO was used to assess the CCC physical activity opportunities and the overall environment to support physical activity. These data were collected by Research Assistants who were trained and observed to achieve 80% inter-rater reliability.

Specifically, in the EPAO physical activity domain (the focus of this manuscript) child behaviors observation category, the following were recorded; the total number of minutes of active play, total number of minutes of structured physical activity, and the number of outdoor active play occasions provided. In the sedentary activity—child observation category, the number of times children were seated ≥30 min was recorded. In the physical activity—staff behavior observation category, documenting number of occasions staff limited active play as a form of punishment, if staff participated in active play with children, number of occasions staff stated positive statements when the children were engaged in physical activity, whether or not staff used prompts to increase or decrease activity, if staff provided formal physical activity education lesson, and if the center offered extra-curricular PA activity programs to children. The center environment category addressed the presence of fixed or portable playground equipment, whether running space was available for children both indoors and outdoors, if there were any space limitations, and identifying if there were any forms of display encouraging physical activity throughout the center and classrooms.

## 3. Statistical Analyses

Descriptive characteristics of the study sample were provided for continuous variables as means and standard deviations, and for categorical variables as counts and percentages. The Wilcoxon rank sum test, Pearson’s Chi-squared test, and Fisher’s exact test were used, depending on the distribution of data, to compare the difference in the demographic variables between the intervention versus control arms within the overall, CWD, and non-CWD groups, respectively.

The primary modeling approach included fitting mixed-effects linear regression models to accommodate the repeated measures and nested structure of the data across multiple childcare centers. The impact of the HC2 intervention versus control on child PBMI and the EPAO physical activity sub-score over one school year (T1 to T2) was examined, with participant ID controlled as the random effect to account for clustering. The primary binary predictor was the randomization arm (HC2 intervention or control), while time and the interaction term of randomization arms × time were also controlled in the model. The time effect was analyzed as a continuous variable. To assess the impact of the intervention arm on child PBMI and CCC EPOA PA items change over time moderated by disability, a three-way interaction term (Time (T1 to T2) × Arms (Intervention vs. Control) × Disability (Yes/No)) was created and included in the final models. To account for the unbalanced distribution of child demographics, all multivariable regression models focusing on child PBMI controlled for child race, ethnicity, and English proficiency.

A *p*-value less than an alpha level of 0.05 was considered statistically significant. All data analysis was performed with R statistical software version 4.3.2 [29].

## 4. Results

Child (total analytical sample, N = 274) demographic information stratified by CWD and experimental condition is summarized in Table 1. Hispanic children were more prevalent in the intervention group compared to the control group (79% vs. 68%, *p* = 0.034), and a higher percentage of children in the control group were proficient in English (70% vs. 52%, *p* = 0.011). Among children without disabilities, the control group had a higher proportion of Black children compared to the intervention group (32% vs. 6.7%, *p* = 0.043) and more Hispanic children in the intervention group compared to the control group (89% vs. 68%, *p* = 0.004). Additionally, a greater percentage of children in the control group were proficient in English compared to the intervention group (84% vs. 33%, *p* < 0.001).

Table 2 data present EPAO PA items and child PBMI and changes over one school year by experimental condition. In the HC2 intervention centers, there was a significant increase in physical activity staff behaviors (from 12.67 to 16.33, *p* < 0.001) and physical activity policy scores (from 9.44 to 15.42, *p* < 0.001) and a significant decrease in child BMI percentile (from 68.57 to 61.56, *p* = 0.007) over one school year. In the control centers, there was a significant increase in sedentary opportunities (from 12.63 to 17.08, *p* < 0.001) and significant decreases in the portable play environment (from 15.19 to 11.96, *p* = 0.03), fixed play environment (from 12.57 to 10.00, *p* = 0.04), and physical activity staff behaviors (from 19.37 to 15.75, *p* < 0.001).

Multilevel models examining the impact of HC2 arms (intervention vs. control), CWD status (yes vs. no), and respective interaction with time (T1 to T2) for PBMI and the EPAO physical activity total score are presented in Table 3. Overall, there were no significant differences in child PBMI and EPAO PA items by experimental condition over one school year. However, CWD status was associated with a significantly higher physical activity total score (β = 7.55, 95% CI: 5.29 to 9.81, *p* < 0.01). There were no significant differences found for child PBMI change over time when comparing CWD versus non-CWD (beta = 1.62 95% CI [−7.52,10.76], *p* = 0.73). Over one school year, change in the EPAO physical activity score was significantly moderated by CWD status (β = −6.27, 95% CI: −7.69 to −4.85, *p* < 0.01).

When the three-way interaction term of randomization arms, CWD status, and time was included in the multilevel model, the results showed CWD status remained significantly associated with higher physical activity total scores (β = 7.31, 95% CI: 4.22 to 10.40, *p* < 0.01), and time continued to show a significant positive effect (β = 1.57, 95% CI: 0.18 to 2.95, *p* = 0.04). Additionally, the interaction between CWD status and time again indicated a significant decrease in the physical activity total score (β = −6.30, 95% CI: −8.30 to −4.29, *p* < 0.01). No significant findings were shown for the same three-way interaction term on child PBMI (*p* = 0.87) and EPAO physical activity (*p* = 0.75).

**Figure 1:**
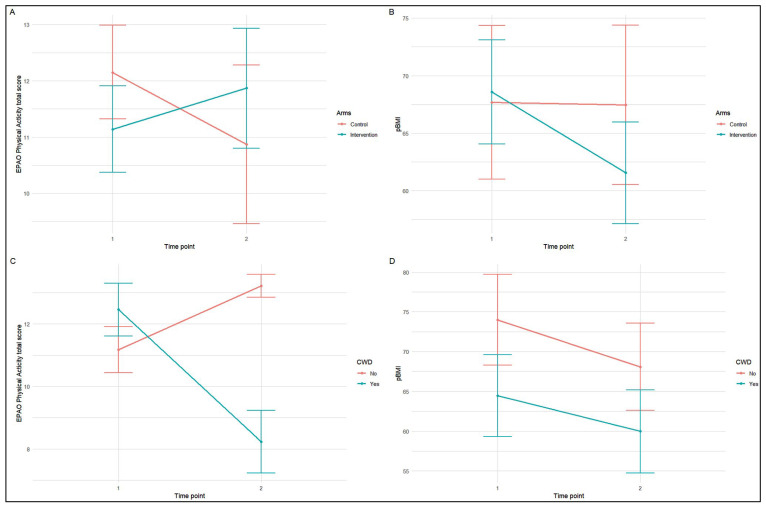
EPAO physical activity total score and child PBMI mean change over 1 school year by randomization arm (**A**,**B**) and by disability status (**C**,**D**).

Figure 1 depicts the mean change in child PBMI and the EPAO physical activity total score from T1 to T2 with a 95% confidence interval by intervention arms (Figure 1A,B) and by CWD status (Figure 1C,D). At HC2 intervention centers, increased total PA scores from T1–T2 were seen. In addition, decreased PBMI at intervention centers was observed with PBMI remaining constant at the control center.

## 5. Discussion

The present study aimed to investigate the impact of the HC2 intervention on change in child body PBMI and the CCC PA environment over one school year versus control CCCs. All CCCs serve CWD so a key interest of this analysis was to explore if there were any differences by child disability status in PBMI change. Findings showed a positive impact of the HC2 intervention on PBMI, highlighting the potential of structured PA programs in promoting healthy weight trajectories in young children, including those with disabilities. No significant differences were found in PBMI changes between CWD and children without disabilities, suggesting the intervention’s efficacy across both groups. At the control centers, the results showed increased sedentary behaviors (times children seated ≥ 30 min), decreased portable play equipment, and decreased fixed play environment, while in HC2 intervention, CCCs PA staff behaviors and positive policy changes increased. The findings presented in this study underscore the premise of implementing evidence-based interventions, such as HC2 to prevent obesity among preschool-aged children with and without disabilities. These results also provide evidence to foster PA and the supporting environment in CCC health promotion and obesity prevention interventions.

### 5.1. Impact of Physical Activity Environment on PBMI

Our first key finding was that the HC2 intervention centers where the curriculum was adapted for CWD exhibited a significant reduction in PBMI (mean change −6.74, *p* = 0.007) over one school year versus control centers (no significant change). HC2 centers included increased opportunities for active play and increased PA in minutes per day as part of the intervention. This suggests that an enriched PA environment, facilitated by structured healthy weight interventions, plays a critical role in managing and potentially preventing obesity risk in early childhood.

The significant changes in PMBI at the intervention centers further confirm previous findings that the HC2 intervention demonstrates effectiveness in maintaining a healthy PBMI among multiethnic children including those with disabilities [24,30]. By focusing on the physical activity environment within childcare centers, the HC2 program targeted high-risk populations, including CWD and racial/ethnic minority children, who are disproportionately impacted by the obesity epidemic [31]. This highlights the potential of the CCC environment as a setting for implementing obesity prevention interventions and promoting healthy lifestyle behaviors from an early age.

According to the Centers for Disease Control (CDC), 5.8% of individuals <65 years of age live with a disability in Miami-Dade County [22]. From the 2016–2020 Miami Matters survey data, 3.5% of children are reported to have a disability [23]. Given these numbers, it is important that interventions used to mitigate obesity in children, also include CWD. A second key finding here was no significant effects were found for child PBMI change over time when comparing CWD versus non-CWD, thus suggesting the HC2 was equally effective for both groups. This is a crucial finding as it demonstrates that inclusive, adapted HC2 strategies implemented in the CCC settings can successfully address PBMI management across diverse populations of children, regardless of disability status. Such findings not only promote equity in healthcare delivery but also emphasize the feasibility and effectiveness of universal health interventions in tackling childhood obesity and related health outcomes. This supports the development of inclusive health policies and practices that cater to the diverse needs of all children, contributing to more equitable health outcomes in broader community settings.

### 5.2. Changes in EPAO PA Components

The American Academy of Pediatrics (AAP) recommends that children ages 3–5 participate in at least three hours of physical activity per day, or about 15 min every hour they are awake [32]. Environments that promote PA for all children could contribute to the potential impact of community interventions for preventing overweight and obesity including CWD [10]. Social and built environments (i.e., policies, food availability, access to physical activity, norms, culture) that encourage people to consume more calories than they expend, leading to obesity, are referred to as obesogenic environments [33,34]. CWD face many challenges and barriers to accessing environments that allow them to engage in PA that positively promotes their development and well-being [35] These obesogenic and unsupportive environments may play a part in the rapid increase in obesity rates seen in CWD [10,34]. Our third key finding was that significant differences were observed in various PA components between the control and intervention centers. The control centers recorded increased sedentary opportunities (*p* < 0.001) and decreased access to portable (*p* = 0.03) and fixed play environments (*p* = 0.04). The implications of these results suggest that the centers receiving the HC2 curriculum implemented the tools provided that encouraged PA among all children, including CWD.

Evidence points to enablers and/or barriers to the successful prevention of obesity in children attending CCC. Key enablers and/or barriers include PA policies and staff development and training [36,37,38]. In contrast to control centers, intervention centers demonstrated significant improvements in PA staff behaviors and policy changes. These findings underscore the importance of a holistic approach in promoting PA. The increase in PA staff behaviors at HC2 intervention centers is a pivotal finding, highlighting the effectiveness of staff training and curriculum implementation in enhancing PA promotion. Staff in HC2 intervention centers were more engaged in facilitating and participating in PA with the children, which not only provided immediate PA opportunities but also modeled active behavior for the children to emulate. Furthermore, the significant changes in PA policies at these centers indicate support at the institutional level.

The overall results here highlight the global public health challenge of childhood obesity, its long-term health implications, and its disproportionate impact on children with disabilities (CWD) and minority populations. The study’s findings not only support the need for inclusive health policies but also highlight the role of CCC environments in obesity prevention and the promotion of healthy lifestyles. The observed improvements in PA staff behaviors and policy changes at HC2 centers further underscore the importance of supportive environments in facilitating physical activity among all children, including those with disabilities. These insights contribute to a broader understanding of how tailored interventions in childcare settings can address health disparities and promote long-term health outcomes for all children [39,40,41].

### 5.3. Limitations

Despite the strengths of the study, several limitations should be acknowledged. Firstly, the study’s sample size and demographic characteristics may limit the generalizability of the findings to other populations. Additionally, the reliance on self-reported data from CCC teachers for some EPAO items introduces a potential bias. Also, while the EPAO instrument provides useful information through a one-day snapshot of a CCC environment and practices, the instrument could be implemented over multiple days to reduce response bias. Lastly, data collection occurred during the COVID-19 pandemic, and although measures were collected as per center policies at the time, this may have impacted overall study findings.

## 6. Implications for Research

The successful implementation of the HC2 intervention highlights its crucial role in addressing the obesity epidemic, particularly among vulnerable populations like children with disabilities (CWD). This approach prioritizes childcare centers (CCCs) as key environments for promoting healthy behaviors, leveraging existing infrastructures to reach a diverse range of children. This inclusive strategy not only supports equity in health interventions but also recognizes the heightened obesity risk faced by CWD, emphasizing the importance of tailored approaches in early childhood settings.

### 6.1. Implications for Early Childhood Obesity Prevention

The results of this study have significant implications for early childhood obesity prevention, particularly for children with disabilities who may face additional barriers to engaging in PA. Implementing structured obesity interventions like HC2 that include PA policies and other components in CCCs can lead to improvements in PA environments to support healthy weight development trajectories in early childhood. Policymakers and educators should consider integrating such programs into early childhood settings to address obesity risk factors from a young age.

### 6.2. Recommendations for Future Research

Future research should prioritize longitudinal studies to assess the sustained impacts of structured physical activity interventions like HC2 on BMI and health outcomes in diverse populations, including children with various disabilities. Comparative studies should investigate the effectiveness of specific intervention components, such as increased active play opportunities and policy changes, in promoting active lifestyles and preventing obesity in childcare settings. Additionally, there is a need to develop inclusive strategies tailored to the needs of children with disabilities, while also examining how environmental modifications and policy adaptations within childcare centers influence obesity prevention efforts. Research should also explore multifaceted approaches that integrate physical activity promotion with nutrition education and parental involvement programs, aiming to reduce health disparities and promote health equity among all children.

## 7. Conclusions

The findings here show the significant impact of the HC2 intervention on promoting healthy weight trajectories among young children, including those with disabilities. The HC2 intervention led to notable improvements in physical activity staff behaviors and policy changes in the CCC environment, and a significant decrease in child BMI percentile over one school year versus control CCCs. Importantly, no significant differences in BMI changes were found between children with and without disabilities, demonstrating the intervention’s efficacy across diverse populations. Conversely, control centers experienced increases in sedentary behaviors and declines in play environments. These findings highlight the critical role of structured PA programs in mitigating childhood obesity, particularly in supportive CCC environments. It will be important for future research to refine inclusive strategies that address the unique needs of children with disabilities, thus fostering equitable health outcomes in early childhood settings.

## Figures and Tables

**Table 1:** Child demographic characteristics by disability status and HC2 intervention or control status.

	Total (N = 274)			Disability (N = 155)			No Disability (N = 102)		
	Control N = 74	Intervention N = 200	*p*-Value ^a^	Control N = 51	Intervention N = 104	*p*-Value ^a^	Control N = 20	Intervention N = 82	*p*-Value ^a^
Age (years), Mean (SD)	3.16 (0.99)	3.04 (1.05)	0.4	3.17 (0.96)	3.04 (1.08)	0.4	3.14 (1.08)	3.03 (1.03)	0.9
Gender, n (%)			0.6			0.8			0.2
Female	32 (52)	85 (49)		20 (48)	48 (51)		12 (63)	36 (47)	
Male	29 (48)	89 (51)		22 (52)	47 (49)		7 (37)	41 (53)	
Race, n (%)			0.6			0.7			0.043 *
White	44 (71)	124 (73)		31 (72)	61 (66)		13 (68)	61 (81)	
Black	13 (21)	24 (14)		7 (16)	19 (21)		6 (32)	5 (6.7)	
Native American	0 (0)	4 (2.4)		0 (0)	4 (4.3)		0 (0)	0 (0)	
Asian Pacific Islander	0 (0)	1 (0.6)		0 (0)	0 (0)		0 (0)	1 (1.3)	
Multiracial	3 (4.8)	6 (3.6)		3 (7)	4 (4.3)		0 (0)	2 (2.7)	
Other	2 (3.2)	10 (5.9)		2 (4.7)	4 (4.3)		0 (0)	6 (8)	
Ethnicity, n (%)			0.034 *			0.4			0.004 *
Hispanic	42 (68)	135 (79)		29 (67)	66 (70)		13 (68)	67 (89)	
Non-Hispanic	5 (8.1)	10 (5.8)		5 (12)	10 (11)		0 (0)	0 (0)	
English Proficiency, n (%)	43 (70)	88 (52)	0.011 *	27 (64)	62 (67)	0.8	16 (84)	25 (33)	<0.001 *
Have Health Insurance, n (%)	62 (100)	139 (94)	0.061	43 (100)	84 (93)	0.2	19 (100)	55 (95)	0.6

^a^ Fisher’s exact test; Pearson’s Chi-squared test; Wilcoxon rank sum test. * *p*-value under 0.05 is considered statistically significant.

**Table 2:** EPAO physical activity and child PBMI change over one school year by experimental condition, respectively.

		Intervention Child Care Centers (N = 5)			Control Child Care Centers (N = 5)		
EPAO Domain		Fall 2021 (T1)	Spring 2022 (T2)	Change *	Fall 2021 (T1)	Spring 2022 (T2)	Change *
		(Mean ± SD)	(Mean ± SD)	(*p*-Value)	(Mean ± SD)	(Mean ± SD)	(*p*-Value)
Physical Activity	Active Opportunities	7.04 ± 3.94	8.19 ± 3.68	1.16 (0.33)	6.85 ± 3.33	5.21 ± 3.21	−1.64 (0.15)
	Sedentary Opportunities	15.19 ± 3.07	16.67 ± 3.41	1.57 (0.12)	12.63 ± 3.06	17.08 ± 3.42	4.45 (<0.001) *
	Sedentary Environment	13.33 ± 5.60	11.11 ± 8.26	−2.22 (0.33)	14.39 ± 4.59	12.92 ± 5.69	−1.47 (0.40)
	Portable Play Environment	8.89 ± 6.34	6.19 ± 3.23	−2.69 (0.05)	15.19 ± 4.86	11.96 ± 3.93	−3.16 (0.03) *
	Fixed Play Environment	10.62 ± 2.20	9.79 ± 3.49	−0.83 (0.38)	12.57 ± 4.05	10.00 ± 3.13	−2.59 (0.04) *
	Physical Activity Staff Behaviors	12.67 ± 3.43	16.33 ± 2.62	3.66 (<0.001) *	19.37 ± 2.75	15.75 ± 3.09	−3.29 (<0.001) *
	Physical Activity Training	11.94 ± 6.67	11.25 ± 6.12	−0.76 (0.17)	10.79 ± 7.12	7.81 ± 7.30	−2.82 (0.18)
	Physical Activity Policy	9.44 ± 6.39	15.42 ± 7.79	6.12 (<0.001) *	5.79 ± 7.69	6.25 ± 9.57	−1.36 (0.24)
	Total Physical Activity score	11.14 ± 1.67	11.87 ± 2.66	0.73 (0.31)	12.15 ± 1.86	10.87 ± 2.88	−1.28 (0.13)
PBMI	Child BMI percentile	68.57 ± 32.57	61.56 ± 31.77	−6.74 (0.007) *	67.68± 29.26	67.46 ± 30.38	−1.35 (0.74)

* A *p*-value under 0.05 is considered statistically significant.

**Table 3:** Mixed effect of linear regression models to estimate the effect of HC2 intervention, CWD status, and Time (T1 to T2) on child PBMI and EPAO physical activity total score.

Model	Effect	Child BMI Percentile		Physical Activity Total Score	
		Beta (95% CI) **	*p*-Value *	Beta (95% CI)	*p*-Value *
1	Intervention vs. Control	4.66 (−11.88, 21.23)	0.58	−3.02 (−6.32, 0.28)	0.08
	Time	−1.83 (−10.05, 6.45)	0.67	−1.28 (−2.81, 0.25)	0.11
	Intervention vs. Control × Time	−3.79 (−13.64, 6.00)	0.45	2.01 (−0.07, 4.08)	0.07
2	CWD vs. non-CWD	−9.51 (−25.01, 5.99)	0.23	7.55 (5.29, 9.81)	<0.01 *
	Time	−5.44 (−12.51, 1.64)	0.13	2.04 (1.18, 2.90)	<0.01 *
	CWD vs. non-CWD × Time	1.62 (−7.52, 10.76)	0.73	−6.27 (−7.69, −4.85)	<0.01 *
3	Intervention vs. Control	3.91 (−25.28, 33.08)	0.80	−1.50 (−4.30, 1.29)	0.32
	CWD vs. non-CWD	−8.36 (−38.80, 22.04)	0.59	7.31 (4.22, 10.40)	<0.01 *
	Time	−1.72 (−16.99, 13.61)	0.83	1.57 (0.18, 2.95)	0.04 *
	Intervention vs. Control × Time	−4.72 (−22.00, 12.51)	0.60	0.78 (−0.97, 2.53)	0.41
	CWD vs. non-CWD × Time	−0.14 (−18.31, 18.04)	0.99	−6.30 (−8.30, −4.29)	<0.01 *
	Intervention vs. Control × CWD vs. non-CWD	−0.76 (−36.42, 34.96)	0.97	−0.38 (−5.12, 4.36)	0.88
	Intervention vs. Control × CWD vs. non-CWD × Time	1.72 (−19.38, 22.81)	0.87	0.49 (−2.44, 3.42)	0.75

* *p*-value under 0.05 is considered statistically significant; ** controlling child race, ethnicity, and English proficiency.

## Data Availability

The data that support the findings of this study are available upon reasonable request. Requests for access to the data should be directed to Ruby Natale at the University of Miami.
